# Artificial Intelligence—The Rising Star in the Field of Gastroenterology and Hepatology

**DOI:** 10.3390/diagnostics13040662

**Published:** 2023-02-10

**Authors:** Madalina Stan-Ilie, Vasile Sandru, Gabriel Constantinescu, Oana-Mihaela Plotogea, Ecaterina Mihaela Rinja, Iulia Florentina Tincu, Alexandra Jichitu, Adriana Elena Carasel, Andreea Cristina Butuc, Bogdan Popa

**Affiliations:** 1 Clinical Hospital, University of Medicine and Pharmacy Carol Davila, 050474 Bucharest, Romania; 2 Gastroenterology Department, Clinical Emergency Hospital of Bucharest, 014461 Bucharest, Romania; 3 Gastroenterology Department, “Dr Victor Gomoiu” Clinical Children Hospital, 022102 Bucharest, Romania

**Keywords:** artificial intelligence, endoscopy, endoscopic ultrasound, CT, gastrointestinal diseases, radiology

## Abstract

Artificial intelligence (AI) is a term that covers a multitude of techniques that are used in a manner that tries to reproduce human intelligence. AI is helpful in various medical specialties that use imaging for diagnostic purposes, and gastroenterology is no exception. In this field, AI has several applications, such as detecting and classifying polyps, detecting the malignancy in polyps, diagnosing Helicobacter pylori infection, gastritis, inflammatory bowel disease, gastric cancer, esophageal neoplasia, and pancreatic and hepatic lesions. The aim of this mini-review is to analyze the currently available studies regarding AI in the field of gastroenterology and hepatology and to discuss its main applications as well as its main limitations.

## 1. Introduction

Artificial intelligence (AI) is a term that covers a multitude of techniques that are used in order to resolve different problems through a method that tries to copy human intelligence [1]. In the short and medium term, there is no doubt that among all the technologies that continue to grow, artificial intelligence is the one that brings the most important contributions to the field of medicine. More specifically, diagnostic imaging techniques will play the most important part in this evolution due to their purpose of analyzing images, which represents a very accessible type of intelligence [1]. In gastroenterology, the imaging modalities that are used for diagnostic and staging purposes are endoscopy, endoscopic ultrasound, radiology, and histopathologic examination.

Automatic analysis of gastrointestinal images is performed by a specific subtype of AI that is called machine learning (ML). ML refers to the ability of computers to learn and improve themselves from experiences [2]. The learning process can be either supervised or unsupervised. The supervised method involves teaching the model using a group of input data that are already associated with the output data. Unsupervised learning, on the other hand, means that the system is self-trained for a certain purpose. While supervised learning is usually used for image classification, unsupervised learning usually involves the detection of clusters or different pattern recognition. There are specific techniques used in supervised learning, specifically, support vector machine (SVM), naïve Bayes, and random forest (RF) [1].

Both supervised and unsupervised techniques can be used in order to teach artificial neural networks (ANNs). ANNs are a branch of ML algorithms that are made of node units, which are organized in successive layers [1]. The concept underlying ANNs is the neural network. In the same way that neurons are able to transmit signals through dendrites and axons, artificial neurons (nodes) are also able to transmit different signals. The difference between the two systems is the fact that, unlike the biological neural network, the artificial one is able to receive other forms of signals in addition to the activation ones.

If an ANN is composed of multiple layers, the system is called a deep neural network (DNN), and using this kind of system, is called deep learning (DL). The first layer of the DNN is represented by the input information, and the last one is represented by the output. The layers between input and output are called hidden layers. This kind of compound layering allows the system to make complex decisions based on simpler information.

DL is capable of changing the frameworks in each particular layer based on representation learning, therefore, providing the output more efficiently. This system has a major advantage, and that is transfer learning [3]. This means that a model that was previously trained and has learned the characteristics of an image in one task might be used for a new task [4].

For some medical specialties, especially the ones that need the interpretation of images, such as dermatology, gastroenterology, radiology, and pathology, AI is anticipated to be a technology of great importance.

The aim of this mini-review is to reveal and describe the implementation of AI in the field of gastroenterology.

## 2. Artificial Intelligence in Gastrointestinal Upper and Lower Endoscopy

AI technologies have a large variety of applications in gastrointestinal endoscopy, therefore, improving both diagnostic and treatment performance.

Currently, AI is used in endoscopy to detect, classify, and assess the histology of colorectal polyps, for wireless capsule endoscopy (WCE), for the evaluation of the esogastric pathology by upper endoscopy, and for image analysis of endoscopic ultrasound (EUS) [3] (Table 1).

## 3. Colorectal Polyps

### 3.1. Polyp Detection

For colonoscopy, there are two implementations of AI, computer-aided polyp detection (CADe) and computer-aided polyp diagnosis (CADx) [5,6]. Both of these techniques have been intensively studied.

Taking into consideration that colonoscopy has limitations, such as bowel preparation, adenoma detection rate (ADR), and even fatigue, the rate of missed polyps during colonoscopy might be as high as 25% [7]. Glòria Fernández et al. analyzed the capability of an automatic method to detect colonic polyps based on the creation of energy maps. Although only 24 videos containing polyps were analyzed, the specificity and sensibility of this technique were as high as 72.4% and 70.4%, respectively [8].

Moreover, recently a prospective randomized controlled study carried out by Pu Wang et al. investigated the effect of an automatic polyp detection system. In this study, 1058 patients were enrolled and randomized to either standard or computer-aided diagnosis colonoscopy. They concluded that the AI system notably increased the ADR and also the mean number of adenomas per patient due to a higher incidence of small adenomas that the AI system was able to find [6].

### 3.2. Polyp Classification

**Figure 1:**
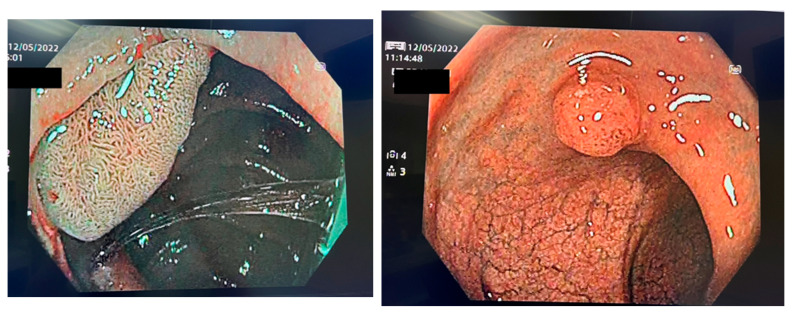
NBI visualization of colon polyps.

The classification of polyps is important, especially when it comes to small ones. This allows the endoscopist to make the best choice and either resect them or leave them in situ. To make these decisions, a close-up analysis using an enhanced imaging technique of the polyps is needed [3]. The final aim of the classification is to determine whether the polyp is malignant or non-malignant. In order to make this differentiation, AI takes into consideration specific characteristics of the polyps, such as the shape, texture, and color [9]. Chromoendoscopy, magnification narrow-band imaging (NBI), endocytoscopy, laser-induced autofluorescence, or confocal endomicroscopy are some techniques used for polyp classification [10,11,12,13] (Figure 1).

Yoshito Takemura et al. performed an analysis concerning narrow-band imaging magnifying colonoscopy to predict the histology of colorectal tumors [14]. Although there is a learning curve in the NBI classification system, and the study was conducted in a single center, the accuracy of this system was nearly 97.8%.

On the other hand, endocytoscopy is able to provide microscopic visualization using mini probes [15]. Yuichi Mori et al. conducted a study regarding the capacity of an automated system for endocytoscopic diagnosis concerning small and diminutive polyps. They found an 89% accuracy of the system for diminutive and small polyps [16].

Laser-induced autofluorescence is a technique that has the capacity to detect colonic dysplasia in vivo [17]. WavSTAT is a device that is incorporated into biopsy forceps and uses laser-induced autofluorescence spectroscopy, allowing laser light to be absorbed by the tissue. The tissue itself emits light; this is further analyzed and represents an optical fingerprint [18].

Chromoendoscopy is a technique in which topical dyes are applied in order to enhance tissue features [19]. This is usually used combined with another technique, such as NBI [20].

Probe-based confocal laser endomicroscopy (pCLE) gives the endoscopist a live microscopic visualization of the epithelial tissue during endoscopy. An automatic software was designed in order to support pCLE, and Barbara André et al. compared its performance with an off-line method used by expert endoscopists. They concluded that both techniques have similarly high sensitivity and specificity [21].

### 3.3. Detection of Malignancy in Polyps

Diagnosing malignancy in polyps is very important because making the right diagnosis guides the optimal treatment for the patients. If deep submucosal invasion is present, surgery is required because there is a high risk of possible metastasis to the lymph nodes [22]. Taking this into consideration, proper endoscopic diagnostic tools should be used in order to be able to use the right therapeutic options.

Endoscopic treatment consists of endoscopic mucosal resection, endoscopic submucosal dissection, or endoscopic full-thickness resection [22,23,24]. Currently, several endoscopic techniques are available to assess the depth of invasion. Those are NBI, high-definition white light endoscopy (HD-WLE), and EUS [25].

Recently, Kenichi et al. evaluated another CAD system for assessing the grade of invasion, which uses ultra-high magnification endocytoscopy [26]. They concluded that this system might be a helpful diagnosing tool in the future, having both high sensitivity and specificity of 98.1% and 100%.

### 3.4. Inflammatory Bowel Disease

When speaking about inflammatory bowel disease, more important than the endoscopic healing of the mucosa is the histologic one. The risk of disease exacerbation and dysplasia is higher when histologic inflammation is still present, and this is harder to assess with conventional colonoscopy, especially in patients who suffer from a type of disease that evolves over several years.

The accuracy of a CAD system was evaluated by Yasuharu et al. using images from colonoscopies of patients with ulcerative colitis. They found a 74% sensitivity and 97% specificity of the system, concluding that it allows the identification of persistent histologic inflammation [27].

Another DNN system was developed by Kento et al. using images of colonoscopies from patients with ulcerative colitis. The system’s accuracy was later tested prospectively on 875 patients with ulcerative colitis who underwent colonoscopy. The accuracy of endoscopic remission was nearly 90.1%, and histologic remission was 92.9% [28].

Regarding Chron’s disease, a retrospective study conducted by Eyal et al. analyzed a deep learning algorithm for the automatic detection of ulcers located in the small intestine using images provided by capsule endoscopy. A convolutional neural network was trained in order to classify the images from the mucosa into either normal or mucosal ulcers. They found an accuracy ranging from 95.4% to 96.7% [29].

### 3.5. Helicobacter Pylori Diagnosis

Helicobacter pylori is a leading cause of gastric cancer. In Asia, the diagnosis of H. pylori by assessing the mucosa is an important part of gastric cancer screening [3]. AI might be a useful tool for improving diagnostic performance taking into consideration that this process is time-consuming and is associated with an abrupt learning curve.

An algorithm that is able to detect H. pylori on specific stained gastric biopsies was designed by Sebastian et al. [30]. They analyzed 87 cases, from which Giemsa-stained biopsies revealed a 100% sensitivity for the algorithm.

### 3.6. Gastritis

Chronic gastritis is an entity that has a high prevalence. It is diagnosed by evaluating the degree of both active and chronic inflammation, assessing the presence of atrophy or intestinal metaplasia, and testing for the presence of Helicobacter pylori infection [1].

A CNN was used in a study conducted by Georg et al. [31]. The capacity of this network was evaluated in order to establish the proper classification of gastritis (autoimmune, bacterial, and chemical). The accuracy of the test was 84% [31].

### 3.7. Gastric Cancer

Gastric lesions are as important as colorectal lesions to be detected early and properly characterized in order to establish optimal treatment options [1]. The gastric lesions that might be premalignant conditions are chronic gastritis and polyps. Several studies analyzed different algorithms that were developed in order to improve the detection of premalignant gastric conditions.

Toshiaki et al. studied the capacity of a CNN system to correctly diagnose gastric cancer lesions with an overall sensitivity of 92.2% [32].

Another study by Yan et al. focused on an artificial intelligence CNN system designed in order to determine the grade of invasion of gastric cancer [33]. They concluded that the system was able to differentiate between early gastric cancer and deep submucosal invasion with a sensitivity and specificity of 76.47% and 95.56%, values that were higher than those achieved by expert endoscopists using standard systems.

Another system that operated on a CNN was developed at the Chinese PLA General Hospital in China and used in order to distinguish between malignant and benign gastric tumors. Their model achieved 100% sensitivity and 80.6% specificity [34].

### 3.8. Esophageal Neoplasia

Esophageal cancer is one of the most aggressive types of cancer. The main histological types are adenocarcinoma and squamous cell carcinoma [1]. Locally advanced esophageal cancer is mainly treated with chemotherapy, and studies demonstrate a positive correlation between histopathological response and the overall rate of survival [35].

One of the most common lesions of the esophagus that is a potential premalignant condition is Barrett’s esophagus. The risk of progression to Barrett’s esophagus increases with the progression of dysplasia. Taking this into consideration, in order to improve prognosis, early detection is of main interest. Nowadays, histopathology represents the gold standard for diagnosing Barrett’s esophagus, although it has limitations regarding interobserver agreement [1]. In order to overcome this limitation, CAD studies that are based on image analysis were recently developed.

Shahriar et al. designed a DL model to help improve the histological diagnosis of dysplasia. Slides from 542 patients were included in the study and were divided into 3 categories: nondysplastic, low-grade dysplasia, and high-grade dysplasia. The model was trained and validated in order to identify dysplasia based on images with an 81.3% sensitivity and 100% specificity for low-grade dysplasia and >90% for nondysplastic Barrett esophagus and high-grade dysplasia [36].

Another retrospective study conducted at the Cancer Institute in Japan used a CNN to detect esophageal cancer early. This system had a 98% sensitivity and was able to distinguish between superficial cancer and advanced esophageal cancer with a 98% accuracy [37].

Regarding Barrett’s esophagus diagnosis, a hybrid ResNet-UNet model was developed by Albert et al. in order to detect neoplasia. The CAD system was able to differentiate and classify the images as either nondysplastic Barrett’s esophagus or as containing neoplasia. Its overall specificity, sensitivity, and accuracy were 88%, 90%, and 89%, respectively [38].

Another aid for helping to determine the grade of dysplasia in Barrett’s esophagus is computerized morphometry. This was used in order to measure several indices of the epithelial nuclei, such as the shape, size, texture, architectural distribution, and symmetry, in a study conducted by Edmon et al. This study proposes, therefore, computerized morphometry as a sustainable tool for determining the grade of dysplasia and predicting the progression to adenocarcinoma [39].

Volumetric laser endomicroscopy is a modern image-based system that has the ability to provide a high-resolution scan of the esophagus’ layers. Although it has high potential to improve the diagnosis of dysplasia in Barrett’s esophagus, its limitations regarding the amount of data needed for real-time interpretations has made it difficult to be used. To overcome this limitation, Anne-Fré Swager et al. designed an algorithm using a clinical volumetric laser endomicroscopy prediction score as an input. The algorithm had a sensitivity and specificity of 90% and 93%, suggesting that an automatic algorithm for detecting early neoplasia has the potential to assist endoscopists [40].

## 4. Wireless Capsule Endoscopy

WCE allows the physician to visualize the small bowel. Although it has high utility by making it possible to diagnose multiple abnormalities, such as mucosal pathology, bleeding, or polyps, WCE has limitations. The most important ones are linked to the large amount of data that needs to be analyzed: nearly 60,000 images and up to 8 h of video in a classic evaluation [3,41].

Taking these limitations into consideration, a study carried out by YuanPu Zheng et al. proved that the detection rate of abnormalities in a classic WCE evaluation is not, in fact, affected by the endoscopist’s experience [42].

At the moment, the software that is used with WCE has the ability to remove image frames that bring no information to the reader and to improve the reader’s efficiency by, for example, using color in order to locate the frames that contain blood.

One of the limitations of the CAD systems that are usually used with WCE is that every time a new application for WCE appears, a new CAD system has to be designed. Santi Seguí et al. developed a system that uses a CNN that reaches almost 96% accuracy for six intestinal motility events [43]. The large number of images that WCE is able to provide can be used to create databases that serve future CAD system development. A study that included 12 endoscopy centers in France retrospectively selected videos from small bowel WCE analysis [44]. They included 4174 videos and extracted from them the ones that contained any pathological findings.

Regarding gastrointestinal bleeding detection by WCE, Xiao Jia et al. designed an automatic bleeding system based on a CNN. They evaluated their method on 10,000 WCE images and found a 99.9% precision value [45]. Yixuan Yuan et al. proposed a novel learning method for the detection of polyps. Their system was based on the idea that images that have similar features should share the same category. The method had a 98% overall accuracy for polyps, bubbles, turbid, and clear images [46].

For the detection of angiectasia, the most common lesion of the small bowel, a CAD system using a CNN was tested with a sensitivity of 100% and a specificity of 96% [44]. Two datasets of still frames were created for algorithm testing and machine learning. Comparable direct learning systems were also reported for the detection of erosions, ulcers, and hookworms [47,48,49].

## 5. Endoscopic Ultrasound

EUS is a useful examination, especially when it comes to diagnosing pancreatic lesions and differentiating chronic pancreatitis from them. Unfortunately, there are limited studies regarding deep learning systems available at the moment.

One study by Maoling Zhu et al. evaluated 262 patients with chronic pancreatitis and pancreatic cancer, respectively. Computer-based techniques were used in order to select texture characteristics from specific regions of interest. A total of 105 characteristics and 9 categories were extracted from the EUS images. The overall accuracy, sensitivity, and specificity of the system were 94.2%, 96.25%, and 93.38% [50].

Ananya Das et al. used digital image analysis on EUS images in order to create a model that has the ability to differentiate between chronic pancreatitis and pancreatic cancer. The analysis was conducted on three groups of patients. One group with normal pancreas, another with chronic pancreatitis, and one with pancreatic adenocarcinoma. Although the number of patients enrolled was small (110 for the normal pancreas group, 99 for the chronic pancreatitis group, and 110 for the adenocarcinoma group), they concluded that direct image analysis of EUS images has high accuracy in differentiating between the three entities [34].

Another aspect that needs to be taken into consideration is the differentiation between autoimmune and chronic pancreatitis. A small study carried out by Jianwei Zhu et al. analyzed 181 cases, 81 with autoimmune pancreatitis and 100 with chronic pancreatitis [51]. They showed that with local ternary pattern variance, textural feature CAD of EUS imaging might be a valuable tool in differentiating autoimmune from chronic pancreatitis.

Another tool that is usefully used combined with EUS in order to differentiate between chronic pancreatitis and pancreatic cancer or to characterize a pancreatic mass is elastography [52]. A cross-sectional study by Săftoiu et al. analyzed the accuracy of EUS combined elastography for pancreatic lesions. A total of 68 patients were included in the study, from which 22 had normal pancreas, 11 had chronic pancreatitis, 32 had pancreatic adenocarcinoma, and 3 had neuroendocrine tumors. Hue histograms of each individual image were calculated by examining the EUS elastography movies. Data were afterward the subject of an extended neural network analysis in order to differentiate benign from malignant characteristics. The sensibility and specificity for differentiating benign from malignant lesions were 91.4% and 97.9 %, respectively, in their study [53].

## 6. Radiology

AI in radiology has become a huge part of diagnostic and therapeutic procedures due to its high applicability among various pathologies.

ML prototypes have been used to process a wide variety of images from computed tomography (CT), magnetic resonance imagining (MRI), and ultrasound (US) [1].

More recently, radiomimics, a term that was first described in 2012, has received great interest due to its capacity to reveal the correlation with biological procedures [54,55].

## 7. AI in Radiology—Applications in Gastrointestinal Pathology

### 7.1. Liver Disease

Metabolic syndrome has a continuous growing curve, replacing the previous most frequent causes of liver cancer, hepatitis and alcoholic cirrhosis [56]. In an article from 2020 that provides an update regarding the incidence of global cancer, liver cancer occupies the third place among the causes of cancer deaths [57]. Taking this into consideration, proper diagnostic tools that provide incipient detection of cancer and proper staging tools need to be used in order to provide the right treatment.

The most frequent liver diseases are represented by hepatocellular carcinoma, non-alcoholic fatty liver disease, benign tumors, viral hepatitis, chronic liver disease, and primary sclerosing cholangitis. For the assessment of liver disease, AI and abdominal US have been used for both diffuse liver disease and focal liver lesions [58]. At the moment, liver biopsy represents the gold standard for the diagnosis of fibrosis and NAFLD. Although it has high sensitivity and specificity, the various complications of this procedure (hemorrhage, peritonitis, pneumothorax) cannot be ignored. Therefore, there is a great need for potential future techniques for diagnosing purposes.

Ilias Gastos et al. proposed to evaluate and classify chronic liver disease using ultrasound shear-wave elastography using a CAD system. A total number of 85 images were analyzed, containing 54 healthy subjects and 31 with chronic liver disease. The accuracy of this model was 87%, with high sensitivity and specificity that reached values of 83.3% and 89.1%, respectively [59].

Regarding the detection and characterization of mass lesions, Schmauch et al. created an algorithm that has the ability to simultaneously realize those two tasks using DL. A total of 367 ultrasound images were used from 367 individuals. The algorithm was attendant by annotations from a radiologist and then tested on 177 subjects. The data reached high receiver operating characteristic curves of 0.93 and 0.916 for lesion discernment and characterization [60].

Koichiro et al. used a DL method with a CNN in order to differentiate between liver masses at CT. A set of liver masses images was used over three phases, non-contrast-agent enhanced, arterial, and delayed. The masses were included under supervised training in five categories: hepatocellular carcinomas, other malignant liver tumors, indeterminate or mass-like lesions, and rare benign liver masses, hemangiomas and cysts. After training, a CNN was tested on 100 liver masses, and the median accuracy of differential diagnosis among liver masses was 0.84 [61].

MRI is also a useful tool for the classification of liver and pancreatic lesions, and several studies evaluated the success of AI when combined with this type of imaging. Fan et al. classified distinct types of liver tissue in patients that were diagnosed with hepatocellular carcinoma using 3-D MRI images and a CNN. Their method was further successfully tested on 20 patients with encouraging results [62].

At the moment, regarding the MRI sequences, only T2-weighted images are used for the automatic grading of liver lesions. Mariëlle conducted a supplementary analysis regarding MRI sequences for automatic classification. Their study included 95 patients, with a total of 125 benign lesions and 88 malignant lesions. DCE-MR and T2-weighted images were analyzed with an overall accuracy of 0.77 [63].

Concerning the potential risk of cirrhosis development by patients with hepatitis, James et al. reported the importance of identifying the viral genetic markers that are mostly associated with the progression of fibrosis. In their study, several sites were identified to correlate with the rate of fibrosis progression using ML techniques, linear projection, and Bayesian networks [64].

Primary sclerosing cholangitis is a liver disease that is characterized by inflammation and fibrosis of the intra and extrahepatic ducts. Moreover, it is a premalignant condition, and effective medical treatment options are lacking. Eaton et al. conducted a study on 509 subjects and estimated the risk and outcomes of the patients with primary sclerosing cholangitis. In order to estimate the risk of disease decompensation, nine variables were taken into consideration: patient age, bilirubinemia, serum alkaline phosphatase, albumin, AST, platelets count, hemoglobin, sodium, and the number of years from the diagnosis. Their tool was able to accurately predict hepatic decompensation by using an ML technique [65].

More recently, AI was used in order to predict graft failure and, therefore, overcome the problems of liver transplantation, such as the high mortality on waiting lists, insufficient donors, and graft failures [66,67,68]. Other factors, such as diabetes, that are associated with death after transplantation were also analyzed using AI [69].

### 7.2. Pancreatic Disease

Several pancreatic diseases were investigated using AI, such as acute pancreatitis and its complications, chronic pancreatitis, pancreatic cystic neoplasms, and pancreatic ductal adenocarcinoma [70,71]. In acute and chronic pancreatitis, AI was used in order to improve disease severity scores and prognostic models. AI was used for the detection, differentiation, and prediction of the malignant potential of pancreatic cystic neoplasms. Pancreatic ductal adenocarcinoma was evaluated using AI by differentiating it from other benign conditions [72]. Moreover, AI is useful in interpreting tissue samples.

Taking into consideration that pancreatic cancer represents the seventh most lethal cancer worldwide and that a five-year survival period depends on the lesion dimensions, the challenge in this pathology is to use proper diagnostic tools in order to provide early detection and to establish the high-risk group of patients [57,73].

Oleg et al. compared different algorithms for risk prediction in pancreatic cancer. Their study included 379 patients, and urine biomarkers (LYVE1, REG1B, TFF1) were analyzed. They set up a biomarker-based risk score that is able to stratify the patients at risk for developing pancreatic cancer [74].

CT is frequently used for screening for the diagnosis of pancreatic cancer, although its sensitivity is not that high, particularly for small lesions [75]. In order to overcome this issue, Liu et al. designed a model of CT scans that was used for the early detection of pancreatic cancer. The model was used on 300 normal scans and 136 pancreatic ductal adenocarcinoma cases and achieved a 90.2% specificity and 80.2% sensitivity [76].

A more sensitive tool for the diagnosis of pancreatic cancer is EUS. Ozkan et al. developed a CAD system for diagnosing pancreatic cancer that uses EUS images [77]. EUS images were extracted from 202 patients with pancreatic cancer and 130 non-cancer patients. Their system reached an 83.3% sensitivity and 93.3% specificity [77].

Another system that uses real-time CAD for pancreatic masses from endoscopic ultrasound imaging was developed by Anca et al. Their system was based on a hybrid convolutional and long short-term memory neural network model. Their study included 65 patients with focal pancreatic masses, and from those, they selected 20 images. The model had a 98.26% accuracy [78].

Acute pancreatitis is a disease in which the outcome depends on its severity. In order to establish the proper treatment and monitoring, acute pancreatitis needs rapid and suitable risk classification. Bodil et al. used ANNs in order to develop a system that is able to predict the severity of acute pancreatitis. A total number of 208 patients were included in their study, and severe pancreatitis was defined relying on the Atlanta criteria. ANNs selected as risk variables the durations of pain, hemoglobin levels, creatinine, heart rate, alanine aminotransferase, and white blood cell count. The system reached 50% sensitivity [79].

Another important pancreatic lesion is represented by pancreatic cystic neoplasms, which are precursor lesions of pancreatic cancer. Their slow progression to invasive carcinoma gives enough time to detect them and to use proper curative treatment. Currently, available technologies that are used in order to establish the risk of cancer are limited [80].

Jayasree et al. retrospectively analyzed pancreatic cysts and parenchyma regions on CT scans from 103 patients in order to predict the risk of intrapapillary mucinous neoplasms (IPMNs). IPMNs were categorized as either low or high risk after resection. Tenfold cross-validation was used combined with clinical variables, obtaining an area under the curve of 0.81 [81].

## 8. Discussion

AI is a promising tool for diagnosis, prognosis, and treatment in the field of gastroenterology and hepatology. Although promising studies have evaluated the specificity and sensitivity of AI systems with promising results, to date, there are only a few devices approved [82]. Some of them are used in the branch of endoscopy, such as EndoBRAIN-EYE, EndoBRAIN, WISE VISION, WavSTAT4, and GI Genius, which are designed in order to detect colon tumors [83,84,85,86]. Moreover, EndoBRAIN-Plus has the possibility to establish tumor depth [83]. CAD EYE and Discovery systems are able to assist the endoscopist in detecting colon polyps, therefore raising the rate of adenoma detection [87].

For CT, Liver AI was designed in order to detect liver lesions. Similar systems are Poseidon and Ultrasound, which are used for ultrasonography [82].

AI has promising applications regarding endoscopy techniques by slowly replacing biopsies in the future, which are currently the gold standard for a large variety of lesions. Moreover, the implementation of ML systems improves the quality of lesion detection in WCE, which is a very laborious but useful technique for the evaluation of the small intestine.

Pancreatic cancer has lately become a very studied pathology due to its high mortality caused by late diagnosis. The only efficient treatment in these cases is surgery, but only about 20% of the patients benefit from it [88]. The available diagnostic tools at the moment are represented by: US, EUS, CT, MRI, and positron emission tomography-CT [89]. Of these, EUS seems to have the highest sensitivity and specificity for detecting pancreatic lesions [90]. The main problem of EUS is that the diagnosis is very reliant on the specialist’s experience. AI helps to overcome this problem by assisting professionals in this field to detect abnormalities. Dumitrescu et al. analyzed the sensibility and specificity of AI in a meta-analysis that included 10 studies [89]. The variation in diagnostic accuracy using AI was not wide in their study compared with the literature, which shows no significant variation. Moreover, EUS seems to have the highest sensitivity in detecting lesions of 3 cm, which represents an important step in early diagnosis. Compared to MRI and CT, which have 67% and 53% sensitivity, respectively, EUS has 94.4% sensitivity [91].

Another aspect that AI addresses is non-variceal upper gastrointestinal bleeding which represents a cause of high mortality. Recently, Ungureanu et al. assessed the use of an ANN for the prediction of mortality in patients that presented with non-variceal upper gastrointestinal bleeding. Their study included 914 patients, and the analysis was performed using the Rockall, Glasgow-Blatchford score and AIM65. Their ANN was able to predict mortality with an accuracy of >95%, which was higher than that of the three scores individually analyzed [92].

The European Society of Gastrointestinal Endoscopy (ESGE) position statement regarding AI, published in October 2022, especially regarding the diagnosis and management of gastrointestinal neoplasia stated that, in order to be implemented in a clinical setting, AI should assure a high-quality standard for both the diagnostic and the treatment of gastrointestinal neoplasia [93]. For the diagnosis of potential lesions, AI should enhance the performance of less experienced endoscopists, not of more experienced ones, therefore, increasing the rate of detection. The ESGE advises against the high expectation of the possibility that AI will replace histopathologic examination of the polyps in the future. AI should not replace histopathologic examination but help endoscopists to make the right decisions concerning colorectal polyps [93]. Moreover, their recommendation for future research is to compare the performance of less experienced endoscopists assisted by AI with that of more experienced ones.

Discussing the limitations of artificial intelligence, the most important ones are that there is still a need for further studies to evaluate its efficiency on a higher number of patients and that implementing CADe systems is expensive, so trial programs are needed before purchasing them.

On 12 December 2022, a study carried out by Ahmad et al. concluded that the polyp detection rate on colonoscopy was significantly higher using CADe. The study was performed by 8 experienced endoscopists in a cancer screening program that included 614 patients who were randomized into either a CADe or control group. Although ADR was not significantly higher using CADe (2.4 versus 2.1 per colonoscopy), the polyp detection rate was higher in the CADe group (85.7% versus 79.7%) [94].

More recently, on the 16th of December 2022, Ladabaum et al. published a study that evaluated a trial result from a CADe program that was used for 3 months in one center, which was the largest one from the study compared to another 5 units that served as control. At the center that used CADe, ADR was 40.1% compared with that of the control sites, which was higher, 41.8% [95]. Taking into consideration that this result was different from that of multiple randomized controlled trials, it may suggest that other factors, such as motivation and training, are also important in the process.

To conclude, there is no doubt that AI might be called “the rising star” of the moment in the field of medicine, providing both physicians and patients with future perspectives regarding diagnosis, prognosis, and treatment decisions, but further studies are still needed.

## Figures and Tables

**Table 1:** Applications of CAD and AI in endoscopic procedures.

**LOWER DIGESTIVE TRACT ENDOSCOPY**	
	Polyp detection
	Polyps classification
	Detection of malignancy in polyps
	Inflammatory bowel disease (ulcerative colitis and Chron’s disease)
**UPPER DIGESTIVE TRACT ENDOSCOPY**	
	Diagnosis of Helicobacter pylori
	Inflammatory gastric disease (autoimmune, bacterial and chemical chronic gastritis)
	Gastric cancer
	Esophageal cancer and premalignant conditions (Barret’s esophagus)
**WIRELESS CAPSULE ENDOSCOPY**	
	Angiectasia
	Polyps
	Erosions/ulcers
	Hookworms
**ENDOSCOPIC ULTRASOUND**	
	Chronic pancreatitis
	Pancreatic cancer
	Autoimmune pancreatitis
	EUS Electrography

## Data Availability

Not applicable.
